# Three dimensional collagen scaffolds promote iPSC induction with higher pluripotency

**DOI:** 10.1007/s13238-016-0321-2

**Published:** 2016-10-11

**Authors:** Qi Gu, He Zhu, Lei Chen, Ling Shuai, Jinhui Fang, Jun Wu, Lei Liu, Wei Li, Jianwu Dai, Jie Hao, Qi Zhou

**Affiliations:** 1 State Key Laboratory of Stem Cell and Reproductive Biology, Institute of Zoology, Chinese Academy of Sciences, Beijing, 100101 China; 2 AIIM Facility, ARC Centre of Excellence for Electromaterials Science, Intelligent Polymer Research Institute, University of Wollongong, Innovation Campus, Squires Way, Fairy Meadow, NSW 2519 Australia; 3 State Key Laboratory of Molecular Developmental Biology, Institute of Genetics and Developmental Biology, Chinese Academy of Sciences, Beijing, 100190 China; 4 College of Life Science, Northeast Agricultural University, Harbin, 150030 China

**Dear Editor**,

Extracellular environment plays a role in regulating stem cell fates and three dimensional (3D) scaffolds can be utilized to mimic the internal environment *in vitro*. Currently, many types of cells have been cultured in 3D conditions but only few studies have focused on reprogramming in a 3D environment. 3D culture systems provide circumstances that can better simulate native conditions which are comprised of distinctive cell morphology, oxygen levels, extracellular matrix secretion and concentration gradients of signaling factors (Keung et al., 2010; Gu et al., 2016).

Herein, we used collagen, the major composition of the extracellular matrix (Di Lullo et al., 2002), that serves as scaffolds to offer porous 3D surrounding to mimic *in vivo* environments (Song et al., 2015) and to explore the role of 3D conditions in reprogramming. In this study, we investigated the effect of 3D collagen scaffolds on the reprogramming of mouse embryonic fibroblasts (MEFs) and pig embryonic fibroblasts (PEFs). MEFs could be successfully converted into mouse induced pluripotent stem cells (iPSCs) in 3D collagen scaffolds. After long time incubation, the results demonstrated that 3D conditions increased reprogramming efficiency with high levels of pluripotency in comparison with the conventional 2D method. Another reprogramming method, nuclear transfer (NT), was also detected with high improved efficiency when using the MEFs from 3D as nuclear donor. In addition, reprogramming inhibitors namely *p21* and B-cell translocation gene 2 (*Btg2*), were suppressed during cultivation in 3D collagen scaffolds.

Our first experiment is to investigate the effects of 3D collagen scaffolds for fibroblast cell viability. MEFs were respectively seeded on 2D cell plates and in 3D collagen scaffolds at the same time. After 5 days culture, the cells in 3D collagen scaffolds were characterized by SEM which showed different patterns from 2D (Fig. 1A). AlarmBlue^®^ cell viability assay showed that MEFs cultured in 3D collagen scaffolds had a better viability than those cultured in 2D (Fig. 1B). To further verify the role of cells culture in different conditions, MEFs were collected for qPCR analysis after 5 days culture. MEFs cultured in 3D collagen scaffolds showed lower expression of senescence markers, *p21* and *Btg2*, compared to the MEFs cultured in 2D (Fig. 1C). The down-regulation of *p21* and *Btg2* might promote the metabolism of G1 phase cells and speed up the cell multiplication (Tirone, 2001). Moreover, *p21* and *Btg2* have been also known as two reprogramming inhibitors, the down-regulation of them might boost reprogramming (Bao et al., 2015).

**Figure 1:**
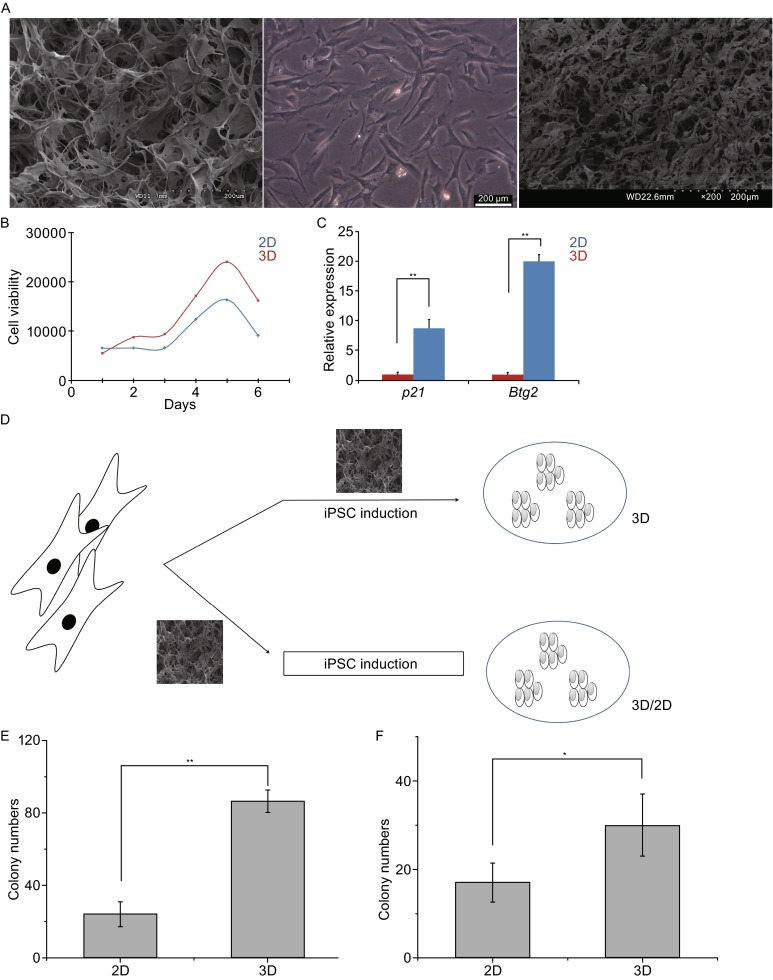
**3D scaffolds improved MEFs growth and mouse iPSC generation**. (A) SEM images of 3D collagen scaffolds (left) and MEFs in 3D collagen scaffolds (right) with 2D control monolayer MEFs (middle). Scale bars = 200 μm. (B) Cell viability analysis for MEFs in 2D and 3D. Y axis number is the value of fluorescence (540/590). (C) qPCR analysis of senescence genes (*p21* and *Btg2*) expression. MEFs in 3D had lower expression of the genes compared to 2D (mean ± s.e.m.; one-way ANOVA, Bonferroni comparison test: **P < 0.001). (D) The scheme of mouse iPSC generation in 3D scaffolds. Up, MEFs were directly induced in 3D scaffolds. Bottom, MEFs were induced on 2D plates after 3D culture. (E) The statistical diagram of colony numbers of 3D and 2D (mean ± s.e.m.; one-way ANOVA, Bonferroni comparison test: **P < 0.01). (F) The statistical diagram of colony numbers of 3D/2D and 2D (mean ± s.e.m.; one-way ANOVA, Bonferroni comparison test: *P < 0.05)

To study the effect of 3D collagen scaffolds on reprogramming, therefore, we used two approaches to reprogram MEFs into iPSCs (Fig. 1D). Firstly, MEFs were grown in 3D collagen scaffolds. Four days later, one part of MEFs was directly reprogrammed in 3D collagen scaffolds (3D), another part of MEFs was digested and seeded on 2D cell plates then reprogrammed (3D/2D). Consequently, the group of 3D and 3D/2D showed a higher efficiency and higher colony numbers compared to those in 2D conditions (Fig. 1E and 1F). When the iPSCs were re-seeded in the 3D scaffolds, the colony formed a grape-like cluster within the pores of 3D collagen scaffolds (Fig. 2A). The limited space on 2D plates inhibited growth of the mouse iPSCs due to the cell-cell connections on the third day whereas the cells grew consistently in 3D scaffolds for at least 6 days (Fig. 2B). The mRNA expression results showed that *Oct4, Zfp42, Gata4, Sox2* and *Klf4* were significantly up-regulated except for *c-Myc* in mouse iPSCs in 3D collagen scaffolds than those cultured on 2D cell plates (Fig. 2C). These results indicated that 3D collagen scaffolds could enhance cell proliferation and stemness of mouse iPSCs. The higher pluripotency demonstrates its future developmental ability (Jiang et al., 2011). Down-regulation of *c-Myc* will reduce risks of tumor formation in grafting experiments (Baudino et al., 2002).

**Figure 2:**
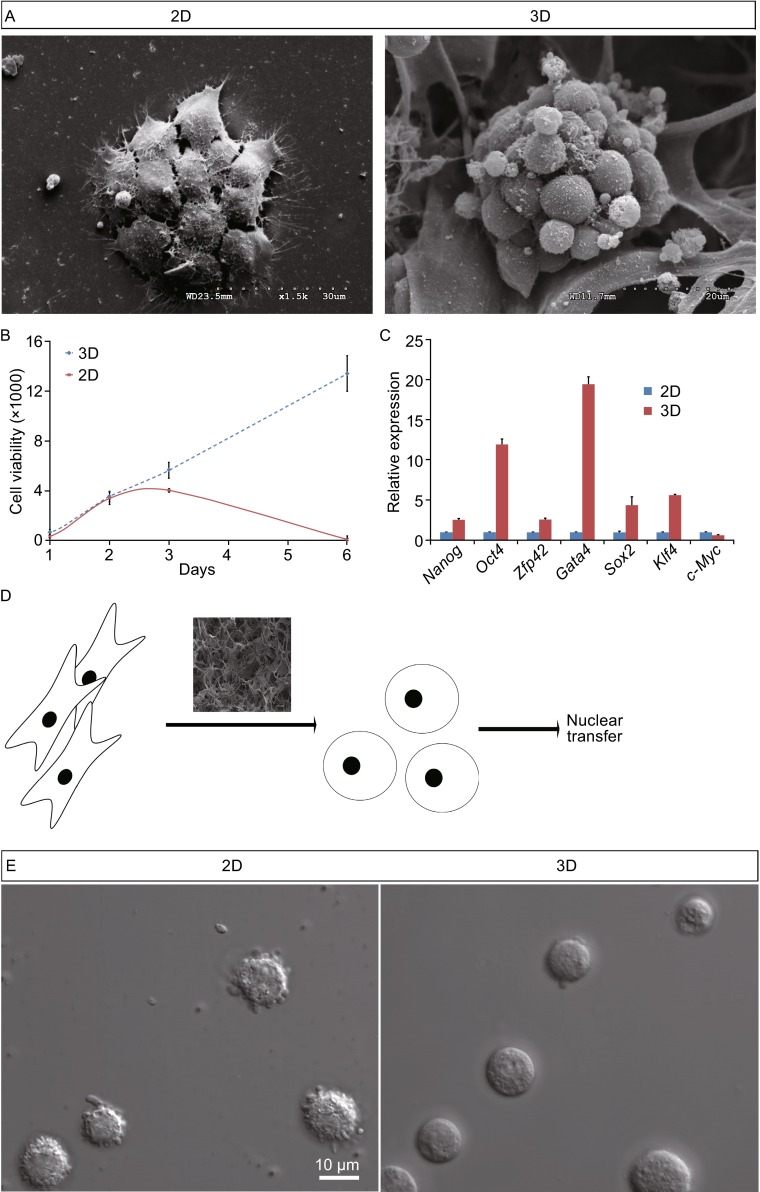
**3D scaffolds used for mouse iPSC and NT**. (A) Scanning images of mouse iPSCs on 2D plates (left) and in 3D scaffolds (right). (B) Cell viability analysis of mouse iPSCs on 2D plates and in 3D scaffolds. Y axis number is the value of fluorescence (540/590). (C) qPCR analysis of pluripotent gene expression for mouse iPSCs on 2D plates and in 3D scaffolds (mean ± s.e.m.). (D) The scheme of NT with donors of MEFs in 3D scaffolds. (E) The single cell status of MEFs on 2D plates and in 3D scaffolds

To further confirm the role of 3D collagen scaffolds in reprogramming, pig iPSCs, commonly difficult to silent their exogenous activation, were derived in 3D conditions from PEFs. PEFs were directly reprogrammed in 3D collagen scaffolds and typical colonies can be observed by SEM (Fig. S1A). Pig iPSCs were cultured in 3D conditions and longer lasting cell viability was observed in 3D compared to 2D conditions (Fig. S1B). The core problem of pig iPSCs is the persistent expression of transgenic genes (Petkov et al., 2015). Our result suggested that the expressions of exogenous, *Oct4* and *c-Myc*, were down-regulated compared to 2D condition (Figure S1C). The endogenous genes of stemness, *Oct4*, *Sox2*, *Rex1* and *Nanog*, were up-regulated when cells were reprogrammed in 3D collagen scaffolds (Figure S1D). The down-regulation of the exogenous genes and up-regulation of the endogenous genes (Fig. S1C and S1D) may bring forward a new approach for reprogramming without Doxycycline (Fujishiro et al., 2013).

Additionally, NT is part of the classic reprogramming methods. NT efficiency is affected by the conditions of the donor cells (Blelloch et al., 2006). In order to understand the influence of 3D culture on NT, MEFs cultured in 3D collagen scaffolds were digested into single cells and served as donors for NT (Fig. 2D). The results were compared and they performed significantly different from 2D group of embryo developments, which included 2-cell, 4-cell, morulae and blastocyst (Table S1). The single cells digested from 3D appeared smooth with no filopodia visible around the edges (Fig. 2E). This suggests that filopodia may not play a role in the proliferation in 3D system, which is different from the present 2D studies (Twarock et al., 2010; Arjonen et al., 2011). During a revision of this work, an independent study (Caiazzo et al., 2016) also reported an iPSCs generation in 3D microenvironments, which supports part of our work. However, we used 3D porous scaffolds whereas they have developed a 3D encapsulation culture.

In summary, the relationship between 3D conditions and stem cell has attracted considerable attentions (Gu et al., 2015). Our study may provide a novel and useful avenue for stem cell research.

## Electronic supplementary material

Below is the link to the electronic supplementary material.

Supplementary material 1 (DOC 233 kb)

## References

[CR1] Arjonen A, Kaukonen R, Ivaska J (2011). Filopodia and adhesion in cancer cell motility. Cell Adhes Migr.

[CR2] Bao X, Wu H, Zhu X, Guo X, Hutchins AP, Luo Z, Song H, Chen Y, Lai K, Yin M (2015). The p53-induced lincRNA-p21 derails somatic cell reprogramming by sustaining H3K9me3 and CpG methylation at pluripotency gene promoters. Cell Res.

[CR3] Baudino TA, McKay C, Pendeville-Samain H, Nilsson JA, Maclean KH, White EL, Davis AC, Ihle JN, Cleveland JL (2002). c-Myc is essential for vasculogenesis and angiogenesis during development and tumor progression. Genes Dev.

[CR4] Blelloch R, Wang Z, Meissner A, Pollard S, Smith A, Jaenisch R (2006). Reprogramming efficiency following somatic cell nuclear transfer is influenced by the differentiation and methylation state of the donor nucleus. Stem Cells.

[CR5] Caiazzo M, Okawa Y, Ranga A, Piersigilli A, Tabata Y, Lutolf MP (2016). Defined three-dimensional microenvironments boost induction of pluripotency. Nat Mater.

[CR6] Di Lullo GA, Sweeney SM, Korkko J, Ala-Kokko L, San Antonio JD (2002). Mapping the ligand-binding sites and disease-associated mutations on the most abundant protein in the human, type I collagen. J Biol Chem.

[CR7] Fujishiro SH, Nakano K, Mizukami Y, Azami T, Arai Y, Matsunari H, Ishino R, Nishimura T, Watanabe M, Abe T (2013). Generation of naive-like porcine-induced pluripotent stem cells capable of contributing to embryonic and fetal development. Stem Cells Dev.

[CR8] Gu Q, Hao J, Lu Y, Wang L, Wallace GG, Zhou Q (2015). Three-dimensional bio-printing. Sci China Life Sci.

[CR9] Gu Q, Tomaskovic-Crook E, Lozano R, Chen Y, Kapsa RM, Zhou Q, Wallace GG, Crook JM (2016). Functional 3D neural mini‐tissues from printed gel‐based bioink and human neural stem cells. Adv Healthcare Mater.

[CR10] Jiang J, Ding G, Lin J, Zhang M, Shi L, Lv W, Yang H, Xiao H, Pei G, Li Y (2011). Different developmental potential of pluripotent stem cells generated by different reprogramming strategies. J Mol Cell Biol.

[CR11] Keung AJ, Kumar S, Schaffer DV (2010). Presentation counts: microenvironmental regulation of stem cells by biophysical and material cues. Annu Rev Cell Dev Biol.

[CR12] Petkov S, Glage S, Nowak-Imialek M, Niemann H (2015). Long-term culture of porcine induced pluripotent stem-like cells under feeder-free conditions in the presence of histone deacetylase inhibitors. Stem Cells Dev.

[CR13] Song T, Zhao X, Sun H, Li X, Lin N, Ding L, Dai J, Hu Y (2015). Regeneration of uterine horns in rats using collagen scaffolds loaded with human embryonic stem cell-derived endometrium-like cells. Tissue Eng Part A.

[CR14] Tirone F (2001). The gene PC3(TIS21/BTG2), prototype member of the PC3/BTG/TOB family: regulator in control of cell growth, differentiation, and DNA repair?. J Cell Physiol.

[CR15] Twarock S, Tammi MI, Savani RC, Fischer JW (2010). Hyaluronan stabilizes focal adhesions, filopodia, and the proliferative phenotype in esophageal squamous carcinoma cells. J Biol Chem.

