# Supplementary material for: Three dimensional collagen scaffolds promote iPSC induction with higher pluripotency
Source: Protein Cell. 2016 Oct 11;7(11):844–8. doi: 10.1007/s13238-016-0321-2 (PMC5084158; doi:10.1007/s13238-016-0321-2)
Supplement: Supplementary file 1 — Supplementary material 1 (DOC 233 kb) [file 13238_2016_321_MOESM1_ESM.doc]

**Supplementary Information**

**Materials and methods**

**Cell culture**

All reagents were purchased from Life Technologies except where indicated. The mouse embryonic fibroblasts (MEFs) were derived from ICR mouse embryos (E13.5) and cultured in 10G medium containing Dulbeco’s Modified Eagle’s Medium (DMEM) supplemented with 10% fetal bovine serum (FBS), 2 mM L-glutamine, 1% nonessential amino acids (NEAA) and 1 000 U/ml penicillin/streptomycin (PS). MEFs were passaged using 0.25% trypsin-EDTA when confluent, and the passage 2 (P2) cells were used for further 2D and 3D culture. The PEFs were derived from Duroc pig embryos (E35) . The isolated embryos were washed in PBS under sterile conditions. After removing the head, limbs, visceral tissues and vertebral columns, the remaining parts were cut into pieces, digested with 0.25% trypsin-EDTA solution for about 4~6 h. After trypsinization, an equal volume of DMEM with 10% FBS was added to stop digestion. Trypsinized tissues were transferred into a new tube, and cells were collected by centrifugation and suspended in a fresh medium.

**Scaffolds preparation**

The collagen scaffolds were a gift from Dai Lab (Institute of Genetics and Development Biology, Chinese Academy of Sciences) and prepared as described previously . Briefly, the collagen membranes were soaked in 0.5 M acetic solution for 8 h at 4^o^C. Then the solution was mixed in a blender for 15 min and neutralized with 4 M NaOH. After that, the solution was dialyzed in deionized water for 5 days and lyophilized. The existing collagen was cut into 1-3 μm thickness, sterilized by Co 60 and stored at 4^o^C before use.

**Reprogramming and nuclear transfer (NT)**

Both mouse and pig iPSCs were derived using retrovirus system previously published . Retroviral vectors (pMX-based) containing murine cDNAs of Oct4, Sox2, Klf4 and c-Myc and the packaging plasmids VSV-G and Gag-Pol were purchased from Addgene. Briefly, these plasmids were transfected into 293T cells using Lipofectamine^TM^ LTX Reagent according to the manufacturer’s instructions. Twelve hours later, the medium was changed with DMEM medium and 1% FBS was added. The virus-containing supernatants were harvested after 36-hour and 60-hour transfection, filtered and concentrated as published previously. MEFs or PEFs in a well of 24-well plate at the density of 10^4^ cells/cm^2^ were infected respectively with concentrated virus-containing solution. At approximately 90% confluency, the cells were split and cultured on feeders. Mouse iPSCs were generated in either 3D (iPSCs derived in 3D scaffolds) or 3D/2D (iPSCs derived on 2D plates with the MEFs wtreated in 3D scaffolds for 4 days) platforms. To compare the difference between 3D and 2D, they were digested into single cells and re-plated onto 2D feeders respectively after 7 days induction. Then, the colonies were counted after another 7 days.

NT was performed as described previously . Matured oocytes were obtained from superovulated female B6D2F1 (C57BL/6·DBA/2) mice (8–10 weeks old). Micromanipulation was performed using the one-step method to reconstruct cloned embryos. MEFs were used as donor cells, the reconstructed embryos were activated for 5 h in an activation medium containing 5 µg/mL cytochalasin B. Cloned embryos were cultured at 37 ^o^C under 5% CO_2_.

**Scanning electron microscopy (SEM)**

The scaffolds with cells were fixed in 2.5% glutaraldehyde reserved for electron microscopy (NOVON, USA) at 4 ^o^C overnight, followed by washing with PBS for three times. Then, the samples underwent dehydration of gradient ethanol, incubated in isoamyl acetate for critical point drying and observed by HITACHI SU8010 SEM (Hitachi Science Systems, Japan). The freeze-dried scaffolds without cells were directly coated with gold and analyzed by SEM.

**Quantitative RT-PCR (qPCR)**

Total RNA was extracted with Trizol LS Reagent and reverse transcription was carried out as previously described . Quantitative PCR analysis was performed in triplicate using the SYBR® Premix EX TaqTM kit (Takara, Japan). Primer sequences were shown in Table S2.

**Test of cell viability**

The constant proliferation of cells was tested by alarmBlue® Cell Viability Assay according to the manufacturer’s instructions. The resulting fluorescence was read using the EnSpire^TM^ Multilabel Reader (PerkinElmer, USA) with 570 nm light.

**Statistical analysis**

In cell proliferation assay and Real-Time PCR experiment, comparative analysis was performed with one-way ANOVA analysis using IBM SPSS software. At least three samples were used for each analysis. Differences between two data sets were considered statistically significant when *p* < 0.05. Error bar was reported in figures as standard deviation.

Table S1 The statistical analysis of NT efficiency

| **Group** | **Reconstructed** | **2-cell (%)** | **4-cell (%)** | **Morulae (%)** | **Blastocyst (%)** |
| --- | --- | --- | --- | --- | --- |
| **3D^a^** | 176 | 119(67.6) | 33(18.8) | 21(11.9) | 11(6.3) |
| **2D^b^** | 141 | 73(51.8) | 18(12.8) | 14(9.9) | 7(5.0) |

*^a,b^* Values with different superscripts are significantly different, *p* < 0.05

Table S2. Primer sequence for Q-PCR

| **Gene name** | | **Forward primers** | **Reverse primers** | | |
| --- | --- | --- | --- | --- | --- |
| ***Mouse*** | | | | | |
| *Oct4* | | TCTTTCCACCAGGCCCCCGGCTC | TGCGGGCGGACATGGGGAGATCC | | |
| *Sox2* | | TAGAGCTAGACTCCGGGCGATGA | TTGCCTTAAACAAGACCACGAAA | | |
| *Nanog* | | TGAGGAGGAGGAGAACAAGGT | ATCTGCTGGAGGCTGAGGTA | | |
| *c-Myc_* | | TGACCTAACTCGAGGAGGAGCTGGAATC | AGTTTGAGGCAGTTAAAATTATGGCTGAAGC | | |
| *Klf4* | | CCATCGGACCTACTTATCTGC | AAAACCTCAAACCAAAACCC | | |
| *Zfp42* | | CCTCACTGTGCTGCCTCCAA | TCCTTCTTGAACAATGCCTATGACTC | | |
| *Gata* | | GACTTCTTCCTTCCTTATTCTC | CCTGCTCAGACTGTAGATG | | |
| *Btg2* | | GGTATGAGCCACGGGAAGAG | TAATGATCGGTCAGTGCGTCC | | |
| *p21* | CATTCAGAGCCACAGGCACC | | | CCATGAGCGCATCGCAATC |  |
|  | |  |  | | |
| *Gadph* | | AGGTCGGTGTGAACGGATTTG | TGTAGACCATGTAGTTGAGGTCA | | |
| ***Pig*** | | | | | |
| *exo-oct4* | | GAGGCCCTTGGAAGCTTAGCC | GAGGCCCTTGGAAGCTTAGCC | | |
| *exo-cmyc* | | CGCTCTGCTGTTGCTGGTGAT | CCCAGTGTGGTGGTACGGGAAATC | | |
| *Oct4* | | CAAACTGAGGTGCCTGCCCTTC | ATTGAACTTCACCTTCCCTCCAACC | | |
| *Sox2* | | CATCAACGGTACACTGCCTCTC | ACTCTCCTCCCATTTCCCTCTTT | | |
| *Rex1* | | ATCCAAGACCACCACCACTG | GTTCACAGCAACATTCAGGTAGA | | |
| *Nanog* | | AATGATCGTCACATATCTTCAGGCTGTA | GTTCCATGGGCTCAGTGGTCAAG | | |
| *β-Actin* | | AGATCGTGCGGGACATCAAG | GCGGCAGTGGCCATCTC | | |


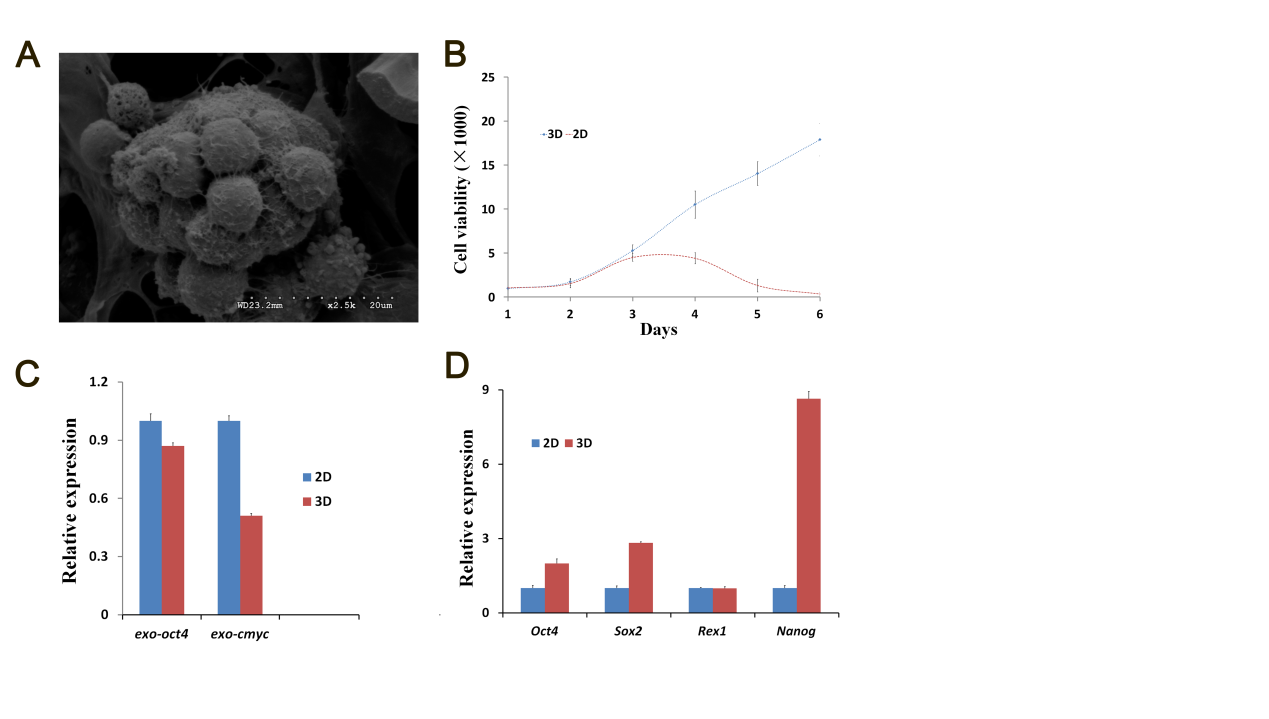


**Figure S1 Pig iPSCs induced in 3D scaffolds**

**A** SEM images of pig iPSCs which were reprogrammed from PEFs in 3D scaffolds.

**B** Cell viability analysis for pig iPSCs on 2D plates and in 3D scaffolds. Y axis number is the value of fluorescence (540/590).

**C-D** qPCR analysis for exogenous gene (C) and endogenous gene (D) expression of pig iPSCs (mean ± s.e.m.).

Reference

Chen, L., Xiao, Z., Meng, Y., Zhao, Y., Han, J., Su, G., Chen, B., Dai, J., 2012. The enhancement of cancer stem cell properties of MCF-7 cells in 3D collagen scaffolds for modeling of cancer and anti-cancer drugs. Biomaterials 33, 1437-1444.

Gu, Q., Hao, J., Hai, T., Wang, J., Jia, Y., Kong, Q., Wang, J., Feng, C., Xue, B., Xie, B., Liu, S., Li, J., He, Y., Sun, J., Liu, L., Wang, L., Liu, Z., Zhou, Q., 2014. Efficient generation of mouse ESCs-like pig induced pluripotent stem cells. Protein Cell 5, 338-342.

Liu, Z., Wan, H., Wang, E., Zhao, X., Ding, C., Zhou, S., Li, T., Shuai, L., Feng, C., Yu, Y., Zhou, Q., Beaujean, N., 2012. Induced pluripotent stem-induced cells show better constitutive heterochromatin remodeling and developmental potential after nuclear transfer than their parental cells. Stem cells and development 21, 3001-3009.

Wang, J., Gu, Q., Hao, J., Bai, D., Liu, L., Zhao, X., Liu, Z., Wang, L., Zhou, Q., 2013. Generation of induced pluripotent stem cells with high efficiency from human umbilical cord blood mononuclear cells. Genomics, proteomics & bioinformatics 11, 304-311.
